# Disseminated Primary Uterine Hepatoid Adenocarcinoma with α-Fetoprotein Production Demonstrated on ^18^F-FDG PET/CT

**DOI:** 10.3390/diagnostics12061447

**Published:** 2022-06-12

**Authors:** Danijela Dejanovic, Marie Boennelycke, Annemarie Gjelstrup Amtoft, Charlotte Birk Christensen, Victoria Wetterstroem, Annika Loft, Trine Jakobi Noettrup

**Affiliations:** 1 Department of Clinical Physiology and Nuclear Medicine, Rigshospitalet, Copenhagen University Hospital, 2100 Copenhagen, Denmark; annemarie.gjelstrup.amtoft@regionh.dk (A.G.A.); annika.loft.jakobsen@regionh.dk (A.L.); 2 Department of Pathology, Rigshospitalet, Copenhagen University Hospital, 2100 Copenhagen, Denmark; marie.boennelycke@regionh.dk; 3 Department of Nuclear Medicine, Copenhagen University Hospital, 2730 Herlev, Denmark; charlotte.birk.christensen@regionh.dk; 4 Department of Imaging and Radiology, Copenhagen University Hospital—North Zealand, 3400 Hil-leroed, Denmark; jenny.victoria.westerstroem@regionh.dk; 5 Department of Oncology, Rigshospitalet, Copenhagen University Hospital, 2100 Copenhagen, Denmark; trine.jakobi.noettrup@regionh.dk

**Keywords:** hepatoid adenocarcinoma, uterus, PET/CT, FDG, AFP

## Abstract

We present the ^18^F-Fluorodeoxyglucose (^18^F-FDG) positron emission tomography/computed tomography (PET/CT) findings in a 57-year-old woman with post-menopausal bleeding diagnosed with hepatoid adenocarcinoma (HAC) with a primary tumour in the uterine corpus and a highly elevated level of serum-α-fetoprotein (S-AFP) at presentation. HAC is a variant of adenocarcinoma with hepatic differentiation representing a heterogeneous group of neoplasms that morphologically and immunphenotypically resemble hepatocellular carcinoma (HCC) but are of extrahepatic origin. Microscopically, they are usually poorly differentiated adenocarcinomas proliferating in solid sheets or in a trabecular or cord-like arrangement. Primary uterine HAC is exceedingly rare with a general poor prognosis, and data is sparse and limited to case reports, making the clinical management challenging. Various primary anatomical sites have been reported in the literature, with the stomach being the most common primary site. ^18^F-FDG PET/CT plays an important role in staging and follow-up in many gynecological malignancies including uterine corpus cancer. To the best of our knowledge, this is the first report describing a primary uterine hepatoid adenocarcinoma with metastases to bone, vagina and lymph nodes on ^18^F-FDG PET/CT. By utilizing the ability of PET to detect early metabolic changes prior to visible structural changes on conventional imaging, this case illustrates a potential role of ^18^FDG-PET/CT in the staging of primary endometrial HAC by depicting distant metastasis that is not readily identifiable on CT alone.

**Figure 1:**
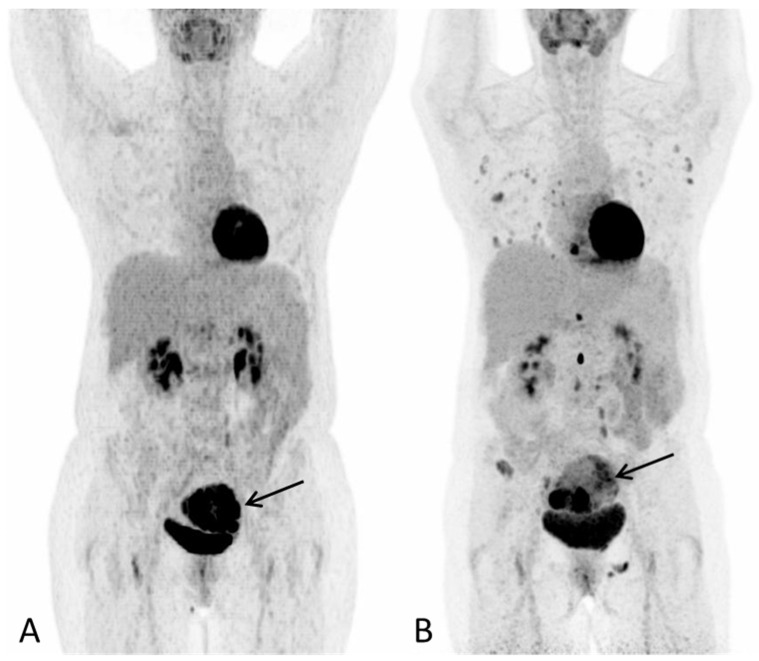
A 57-year-old post-menopausal woman underwent gynecological examination due to intermittent vaginal bleeding over the course of one month with no other symptoms. On transvaginal ultrasound, a tumor thought to represent a leiomyoma was reported. A subsequent hysteroscopy showed the uterine cavity to be filled with soft necrotic tissue, suspected to derive from a leiomyoma. However, histological examination depicted hepatoid adenocarcinoma. At the time of diagnosis, the laboratory work-up for tumor markers showed S-AFP 110610 (ref: <12 kIU/L), Ca-125 13 (ref: <35 kU/L), CA 19-9 17 (ref: <37 kU/L) and CEA 4 (ref: <5µg/L). ^18^F-FDG PET/CT was performed for staging purposes. (**A**) PET Maximum intensity projection (MIP) baseline scan and (**B**) PET MIP evaluation scan after three series of chemotherapy (paclitaxel and carboplatin) showed progression with multiple new metabolic active lesions in the lungs, bones and lymph nodes. The primary tumour in the uterus is seen with increasing inhomogeneous metabolic activity, with areas of low metabolic activity indicating necrosis ((**A**,**B**), arrow). Notably, there were no FDG-avid lesions in the liver on either scan. S-AFP had risen to 258,900 IU/L. Due to progression, the treatment plan changed to sorafenib, which is one of the few systemic treatment options available for advanced HCC.

**Figure 2:**
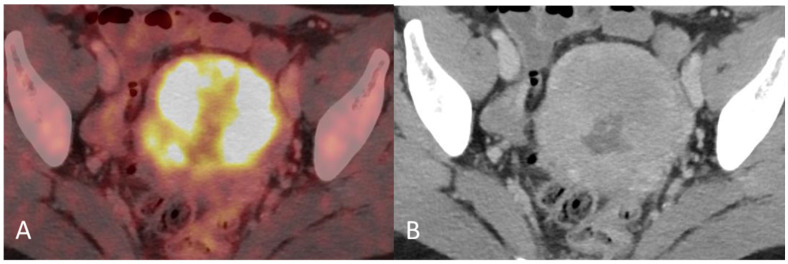
(**A**) Baseline axial PET/CT fused image. The metabolic activity is inhomogeneous, with areas of high to low metabolic activity. (**B**) Contrast-enhanced axial CT shows an enlarged uterus with endometrial thickening and polypoid tumour masses protruding into the uterine cavity. The tumor appears hypodense to the myometrium with signs of myometrial invasion. There was no involvement of the ovaries, fallopian tubes or cervix on either the PET or CT.

**Figure 3:**
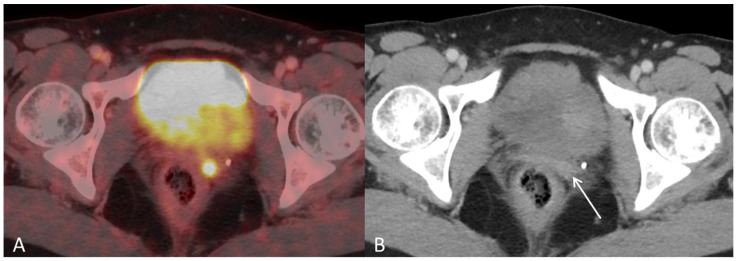
(**A**) Baseline axial PET/CT fused and (**B**) axial CT shows a small metabolic active hyperdense tumour located in the left vaginal corner, verified by biopsy as representing a metastasis from endometrial hepatoid adenocarcinoma ((**B**), arrow).

**Figure 4:**
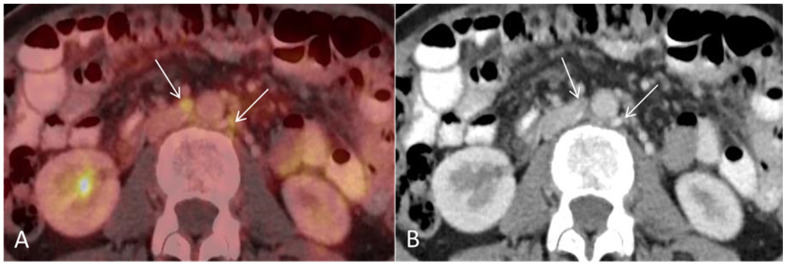
(**A**) Baseline axial PET/CT fused and (**B**) axial CT shows several non-enlarged retroperitoneal lymph nodes with high metabolic activity ((**A**,**B**), arrows). The lymph nodes were not suspicious for malignancy on CT alone.

**Figure 5:**
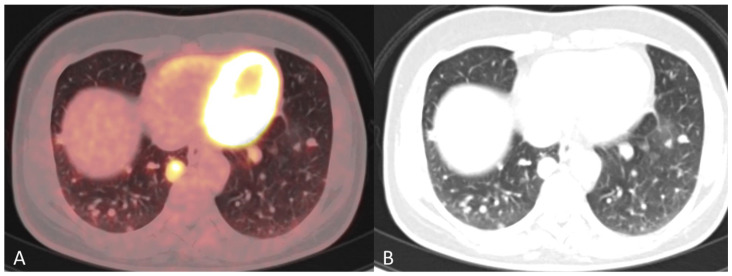
(**A**) Follow-up axial PET/CT fused and (**B**) axial CT after three series of chemotherapy shows progression with multiple metastases in both lungs.

**Figure 6:**
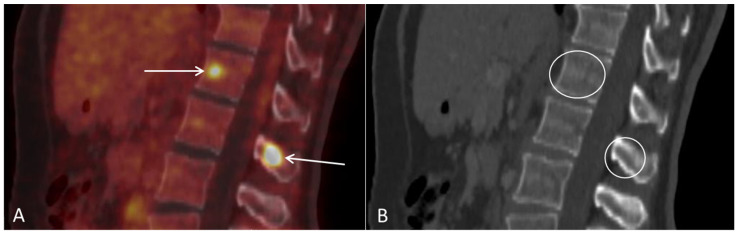
(**A**) Follow-up sagittal PET/CT fused and (**B**) sagittal CT after three series of chemotherapy shows metabolic activity in the spine suspicious for bone metastases ((**A**), arrows), with no corresponding lesions on CT ((**B**), circles).

**Figure 7:**
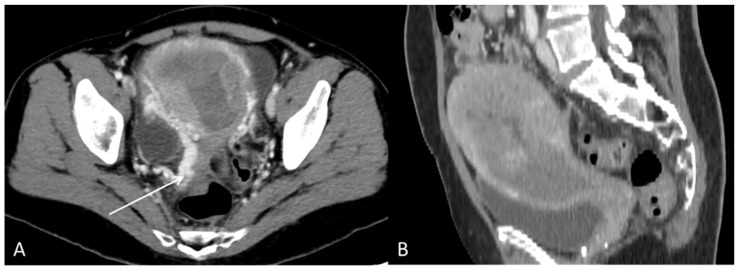
The CT with contrast enhancement performed after approximately two months of sorafenib treatment showed a morphological progression of the primary tumour in the uterus. During this time, the patient had also received radiation therapy (3Gyx10) of the uterus as palliative treatment due to heavy vaginal bleeding. (**A**) Axial CT and (**B**) sagittal CT showed tumour masses in the uterine cavity with patchy hypodensities consistent with necrosis. In the tumour periphery, contrast-enhanced varicose veins were visible, consistent with pelvic congestion syndrome ((**A**), arrow).

**Figure 8:**
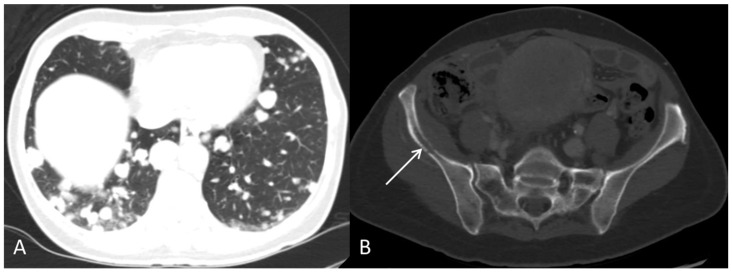
The contrast-enhanced CT, after approximately two months of sorafenib treatment, showed progression with multiple pulmonary metastases (**A**) and an osteolytic metastasis with corticalis destruction in the right pelvic bone ((**B**), arrow). S-AFP was 412,200 kIU/L. Sorafenib was discontinued due to progression. The patient passed away shortly after.

**Figure 9:**
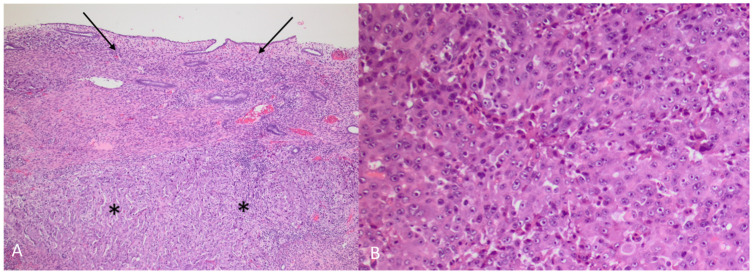
(**A**) HAC (asterisks) growing under intact endometrium (arrows) (Hematoxylin and eosin, 5×) (**B**) HAC (Hematoxylin and eosin, 10×). The tumour was a poorly differentiated adenocarcinoma, primarily growing in solid sheets under a non-neoplastic endometrium. The morphology was not characteristic, and a vague trabecular pattern could only be recognized focally. The tumour cells were polygonal with enlarged, round nuclei and prominent nucleoli and with an abundant cytoplasm. There were numerous mitoses and apoptosis. Prominent blood vessel invasion was present (not shown), as previously reported by others [1,2]. Immunhistochemical staining showed an almost diffuse positive reaction in the tumour cells for AFP (α-fetoprotein) and glypican 3, with a smaller proportion of tumour cells being positive for arginase1 and HepPar1, leading to the diagnosis of HAC. The term HAC was first introduced in 1985 when gastric carcinoma with hepatic differentiation and α-fetoprotein (AFP) production was described [3]. HAC can arise from multiple other organs [4]. In a review of 261 HAC published cases, primary endometrial HAC was found to represent 4% of the documented sites that included lung (5%), gallbladder (4%), pancreas (4%), urinary bladder (3%) and ovary (10%), with stomach (63%) being the most common site [4]. The rarer sites of origin (<2%) were the retroperitoneum, oesophagus, colon, kidney, thymus, fallopian tube and adrenal glands, with the majority constituting single patient cases [4]. Most HAC tumours produce α-fetoprotein (AFP), and AFP serum levels are typically elevated at diagnosis [3,5,6,7]. The high level of AFP secretion during the foetal life, mainly by the liver and yolk sac, is dramatically reduced at birth [8]. An increase of serum AFP after birth is often associated with HCC, yolk sac tumours and benign liver diseases, such as hepatitis [4,8,9]. Although AFP secretion by HAC is a characteristic feature for this group of tumours, there are reports of HAC without AFP production [10,11]. Conversely, there are reports of AFP producing endometrial adenocarcinoma without apparent hepatoid differentiation [12,13]. Other primary uterine neoplasms such as carcinosarcoma and papillary adenocarcinoma have been shown, in rare cases, to have an AFP-secreting hepatoid component [14,15,16,17]. S-AFP has a half-life time of four to six days and constitutes a convenient tumour marker in HAC for diagnosis, evaluation during treatment and detection of recurrent disease. Other tumour markers like CEA, CA-125 and CA 19-9 may vary from normal to slightly elevated in primary uterine HAC [12,13,18,19,20]. Primary endometrial HAC is most common in elderly post-menopausal women with a median age of 64 years at the time of diagnosis [4,15]. The most common symptom is abnormal vaginal bleeding [2,7,18,19,21]. Most tumours are histologically high-grade, with an advanced clinical stage at presentation with a typically aggressive clinical course [15]. Microscopically, they are poorly differentiated adenocarcinomas proliferating in solid sheets or in a trabecular or cord-like arrangement, sometimes with microglandular or canalicular areas. Prognosis is poor, with a reported median survival of eight months [4]. Endometrial HAC metastasis has previously been reported in lymph nodes, lungs and the cervix [2,6,7,19,22].
